# Synthesis, Thermal and Mechanical Properties of Fully Biobased Poly (hexamethylene succinate-*co*-2,5-furandicarboxylate) Copolyesters

**DOI:** 10.3390/polym15020427

**Published:** 2023-01-13

**Authors:** Chengqian Wang, Mingkun Chen, Zhiguo Jiang, Zhaobin Qiu

**Affiliations:** State Key Laboratory of Chemical Resource Engineering, Beijing University of Chemical Technology, Beijing 100029, China

**Keywords:** biobased polyesters, thermal property, mechanical property

## Abstract

Poly (hexamethylene succinate) (PHS) is a biobased and biodegradable polyester. In this research, two fully biobased high-molecular-weight poly (hexamethylene succinate-*co*-2,5-furandicarboxylate) (PHSF) copolyesters with low hexamethylene furandicarboxylate (HF) unit contents (about 5 and 10 mol%) were successfully synthesized through a two-step transesterification/esterification and polycondensation method. The basic thermal behavior, crystal structure, isothermal crystallization kinetics, melting behavior, thermal stability, and tensile mechanical property of PHSF copolyesters were studied in detail and compared with those of PHS. PHSF showed a decrease in the melt crystallization temperature, melting temperature, and equilibrium melting temperature while showing a slight increase in the glass transition temperature and thermal decomposition temperature. PHSF copolyesters displayed the same crystal structure as PHS. Compared with PHS, PHSF copolyesters showed the improved mechanical property. The presence of about 10 mol% of HF unit increased the tensile strength from 12.9 ± 0.9 MPa for PHS to 39.2 ± 0.8 MPa; meanwhile, the elongation at break also increased from 498.5 ± 4.78% to 1757.6 ± 6.1%.

## 1. Introduction

Poly (hexamethylene succinate) (PHS) is a biodegradable aliphatic polyester, which can be synthesized from succinic acid (SA) and 1,6-hexanediol (HDO) through a two-stage melt polycondensation method. In recent years, PHS has been investigated in detail [1,2,3,4]. Yang et al. studied the basic thermal behavior of PHS. They reported that PHS had a glass transition temperature (*T*_g_) of −47.4 °C. PHS exhibited double-melting endotherms due to the melting, recrystallization, and remelting mechanisms [5]. Gesti et al. studied the crystal structure and enzymatic degradation behavior of PHS. The crystal structure of PHS was determined as a monoclinic unit cell, with *a* = 1.612 nm, *b* = 1.464 nm, *c* = 1.440 nm, and *β* = 38.6 °C. They also studied the relationship between enzymatic degradation and the crystal domain of PHS [3]. Franco et al. studied the crystallization kinetics of PHS. Based on the Lauritzen and Hoffman secondary crystallization theory, the nucleation rate parameters (*K*_g_^III^ and *K*_g_^II^) and the transition temperature of the crystallization regime were calculated [4,6]. In addition, some PHS-based copolyesters and nanocomposites have also been reported in the literature [5,7,8,9,10,11,12]. For instance, in a previous work, we synthesized poly (hexamethylene succinate-*co*-ethylene succinate) with 3 mol% of ethylene succinate unit (PHSE3) copolyesters with different molecular weights (12,000 and 58,000 g/mol) and studied the influence of the molecular weight on the crystallization behavior of PHSE3 [5]. Due to the lower melt viscosity and the higher mobility of the polymer chains, the crystallization of low-molecular-weight PHSE3 occurred more easily than that with high molecular weight. Therefore, the melting temperature (*T*_m_) and melt crystallization temperature (*T*_cc_) of the high-molecular-weight polyester decreased during the nonisothermal crystallization; moreover, the crystallization half-time of PHSE3 with high molecular weight increased during the isothermal crystallization. In a previous study, we also synthesized poly (hexamethylene succinate-*co*-butylene succinate) with 6 mol% of butylene succinate (BS) unit (PHSB6) and studied the thermal properties and crystallization behavior [11]. The introduction of the BS unit did not change the crystal structure of PHS; however, the crystallization ability of PHSB6 decreased.

Unlike PHS, poly (hexamethylene 2,5-furandicarboxylate) (PHF) is an aliphatic and aromatic polyester, which can be synthesized from HDO and 2,5-furandicarboxylic acid (FDCA) or its diester through a two-stage melt polycondensation process [13,14,15,16]. As both HDO and FDCA can be derived from biomass, such as fructose and glucose [17,18], PHF may also be regarded as a biobased polyester, although it is not a biodegradable polymer. Similar to terephthalic acid (TPA), FDCA is a dicarboxylic monomer with a rigid cyclic structure [17,19]. Due to the rigid furan ring, the FDCA-based polyesters show a better performance on the thermal and mechanical properties, compared to those of aliphatic polyesters. PHF has a *T*_g_ of about 14 °C and a *T*_m_ of around 145 °C with a heat of fusion (Δ*H*_m_) of 41.2 J/g [20]. Additionally, PHF displays a better crystallizability than poly (ethylene 2,5-furancoate) (PEF) and poly (butylene 2,5-furandicarboxylate) (PBF) due to the long chain flexibility [21,22,23,24,25,26]. Wu et al. reported that PHF had good mechanical properties, with a Young’s modulus of 666 MPa, a tensile strength of 30 MPa, and an elongation at break of 237% [16].

To the best of our knowledge, the synthesis and physical properties of poly (hexamethylene succinate-*co*-2,5-furandicarboxylate) (PHSF) copolyesters have not been studied and reported so far in the literature. Therefore, we synthesized two PHSF copolyesters with low hexamethylene succinate 2,5-furandicarboxylate (HF) unit contents of about 5 and 10 mol% and further investigated the effects of low HF unit contents on the thermal and mechanical properties of PHS. As all the monomers may be derived from renewable resources, PHSF copolyesters may be regarded as fully biobased polymers. The motivation and novelties of this research were as follows: First, the new aliphatic–aromatic PHSF copolyesters with low HF unit contents were synthesized for the first time. Second, PHSF copolyesters were fully biobased polymers from a viewpoint of sustainability. Third, the effect of low HF unit contents may bring remarkable improvements to the physical properties of PHS, especially in the thermal, mechanical, and barrier properties, which should be helpful for achieving a better understanding of the structure and properties relationship in the field of biobased polymers.

## 2. Experimental Section

The raw materials information, including both the monomers, SA and HDO, as well as dimethyl furan-2,5-dicarboxylate (DMFD) and the catalyst tetrabutyl titanate (TBT), is shown in the Supporting Information.

The detailed synthesis procedure of PHS and PHSF is described in the Supporting Information. Scheme S1 displays the synthesis route. For brevity, the two PHSF copolyesters were abbreviated as PHSF5 and PHSF10, respectively, with the number indicating the molar ratio of HF unit.

The structure and properties were characterized with various instruments. For brevity, the details of the characterization section are shown in the Supporting Information.

## 3. Results and Discussion

### 3.1. Chemical Structure and Molecular Weight Studies

**Figure 1:**
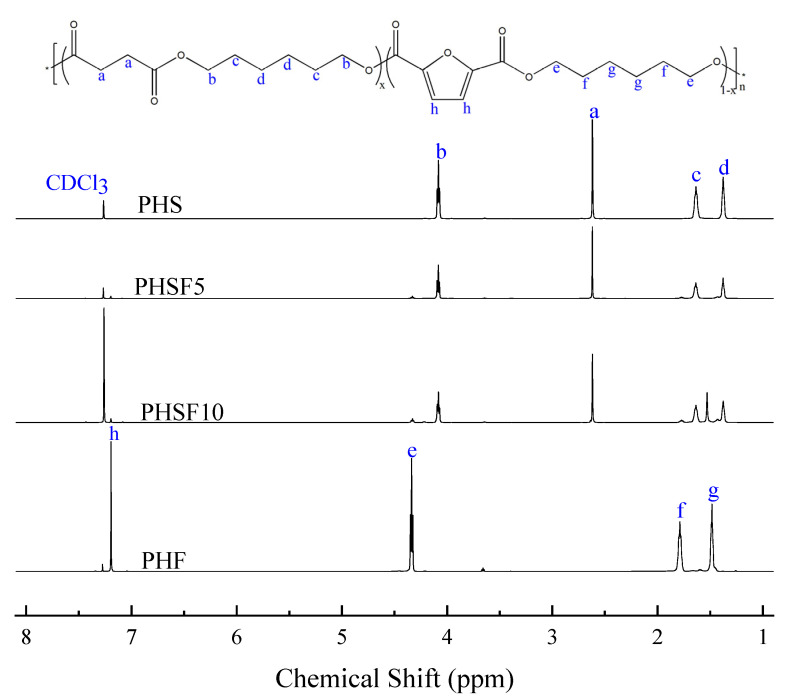
Chemical structure and ^1^H NMR spectra of PHS, PHF, and the copolyesters.

The chemical structure and actual composition of PHSF copolyesters were characterized with hydrogen nuclear magnetic resonance (^1^H NMR). The related spectra of the PHS, PHF, and PHSF copolyesters are illustrated in Figure 1. The NMR result of PHF was cited from our previous study [13]. As demonstrated in Figure 1, the peak at 2.62 ppm (*a*) was from SA, while the peaks appearing at 4.08 (*b*), 1.64 (*c*), and 1.38 (*d*) ppm were attributed to the methylene of the hexamethylene succinate (HS) unit in PHS. For PHF, the peak at 7.19 ppm (*h*) was attributed to the furan ring, while the peaks at 4.34 (*e*), 1.79 (*f*), and 1.48 (*g*) ppm belonged to the methylene of the HF unit. For the PHSF copolyesters, all the signals from the two homopolymers were observed. Moreover, the intensities of the signal from proton *a* gradually decreased with an increase in the HF unit content. Therefore, all the above results indicated that the PHSF copolyesters were successfully synthesized. In addition, the actual composition of the HS unit was calculated via the following equation:

(1) $$\Phi_{\mathrm{HS}\;}=\frac{2I_{h}}{{2I}_{h}{+I}_{a}}$$

where *I*_h_ and *I*_a_ are the integrations of the peaks belonging to the protons (*h*) and (*a*), respectively. As demonstrated in Table 1, the ratio of HS/HF was very close to the feed ratio of SA/DMFD, indicating the successful synthesis of PHSF copolyesters.

The average molecular weights of the PHS and PHSF copolyesters were measured. From Table 1, all samples showed relatively high molecular weights, with the *M*_n_ values being higher than 40,000 g/mol and the *M*_w_ values being higher than 67,000 g/mol. In addition, the PDI values were close to each other.

### 3.2. Crystal Structure Study

**Figure 2:**
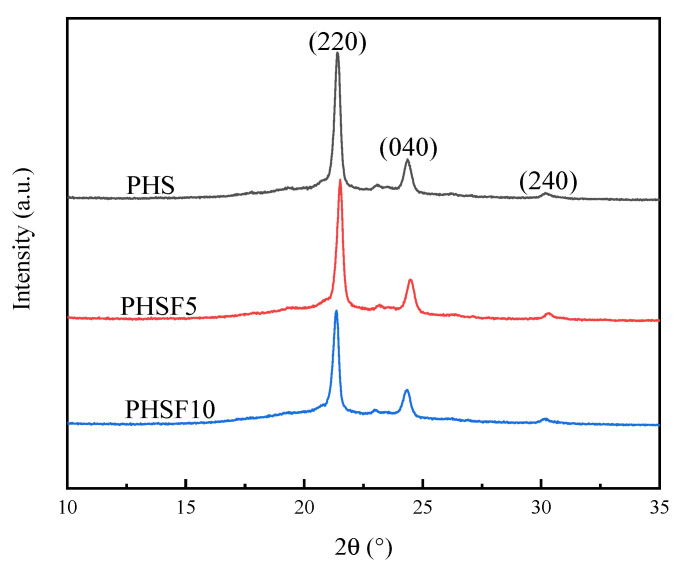
WAXD patterns of PHS and PHSF copolyesters.

Figure 2 displays the wide-angle X-ray diffraction (WAXD) profiles of the PHS and PHSF copolyesters after crystallizing at 32 °C for 10 h. For the PHS homopolyester, three main diffraction peaks appearing at 2θ = 21.3°, 24.2°, and 30.1° were attributed to the (220), (040), and (240) planes of the PHS crystals, respectively [3]. In addition, the PHSF copolyesters showed similar WAXD patterns as PHS, demonstrating that the presence of the HF unit did not change the crystal structure. Therefore, the HF unit should reside in the amorphous region and be expelled from the crystal lattice of the PHS crystals. From Figure 2, the crystallinity values of PHS, PHSF5, and PHSF10 were measured to be about 28%, 25%, and 20%, respectively, indicating that the presence of a small amount of HF unit decreased the crystallinity of PHS to some extent.

### 3.3. Basic Thermal Parameters Study

**Figure 3:**
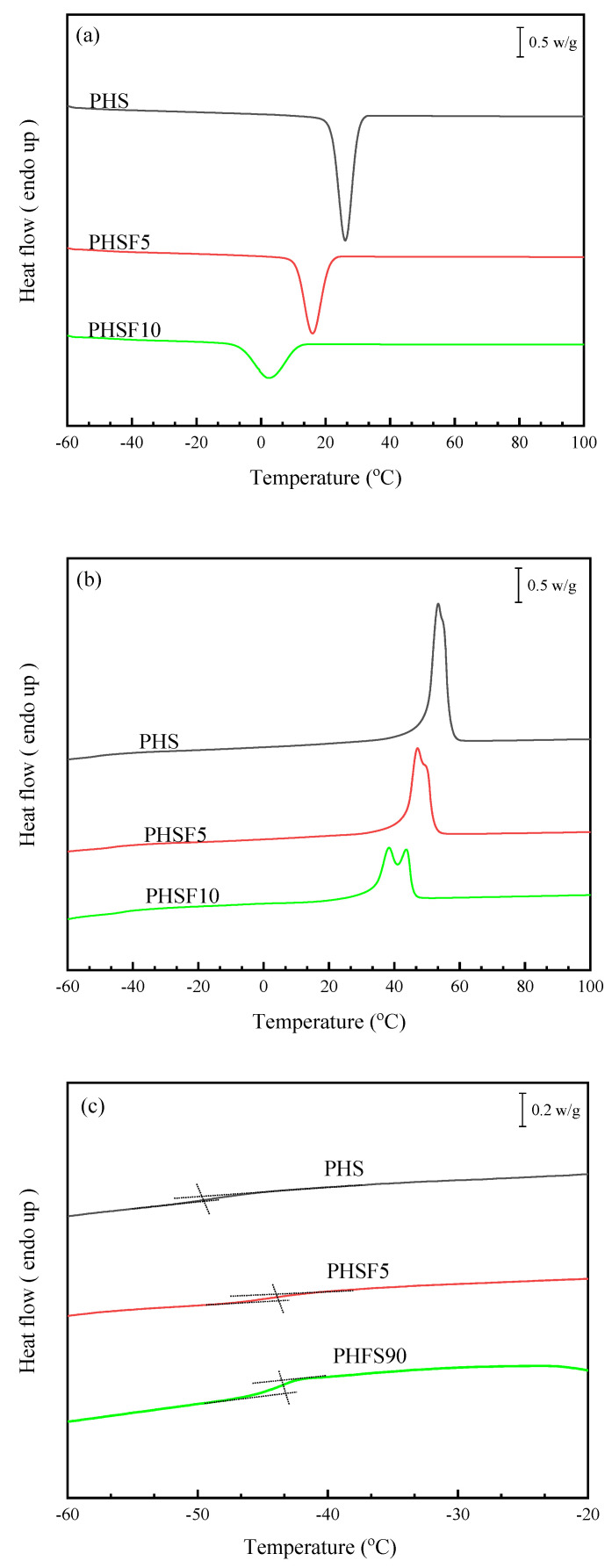
(**a**) DSC cooling traces at 10 °C/min, (**b**) subsequent heating traces at 10 °C/min, and (**c**) DSC heating traces at 20 °C/min after quenching at 60 °C/min from the crystal-free melt of PHS, PHSF5, and PHSF10.

The effect of the HF unit on the basic thermal behavior of PHS was investigated with differential scanning calorimetry (DSC) in this section. Figure 3 depicts the thermal behavior of PHS and its copolyesters, including glass transition, melting behavior, and melt crystallization behavior. From Figure 3a, the *T*_cc_ of PHS was 26.1 °C. With the increasing HF unit content, the *T*_cc_ significantly decreased to 15.9 °C for PHSF5 and 2.4 °C for PHSF10, respectively. Moreover, the melt crystallization enthalpy (Δ*H*_cc_) decreased from 66.2 J/g for PHS to 49.4 J/g for PHSF5 and 37.6 J/g for PHSF10, respectively, indicating again that the crystallinity of PHS was suppressed by the presence of the HF unit. From Figure 3b, PHS displayed a *T*_m_ of 53.4 °C, with a ∆*H*_m_ of 68.3 J/g. However, the *T*_m_ and ∆*H*_m_ of the PHSF copolyesters gradually decreased with an increasing HF unit content because the regularity of the molecular chain of PHS was disturbed by the HF unit. Moreover, as shown in Figure 3b, double-melting endothermic peaks were observed for all samples during the heating process. With an increasing HF unit content, double-melting endothermic peaks became more obvious. Such a phenomenon may well be explained by the melting, recrystallization, and remelting mechanism [27,28,29]. Figure 3c depicts the enlarged glass transition region. From Figure 3c, PHS had a very low *T*_g_ of −48.8 °C, while the *T*_g_ of the PHSF copolyesters slightly increased because the presence of the furan ring decreased the chain mobility. The relevant basic thermal parameters of the PHS and PHSF copolyesters are listed in Table 2 for comparison.

### 3.4. Isothermal Crystallization Kinetics Study

**Figure 4:**
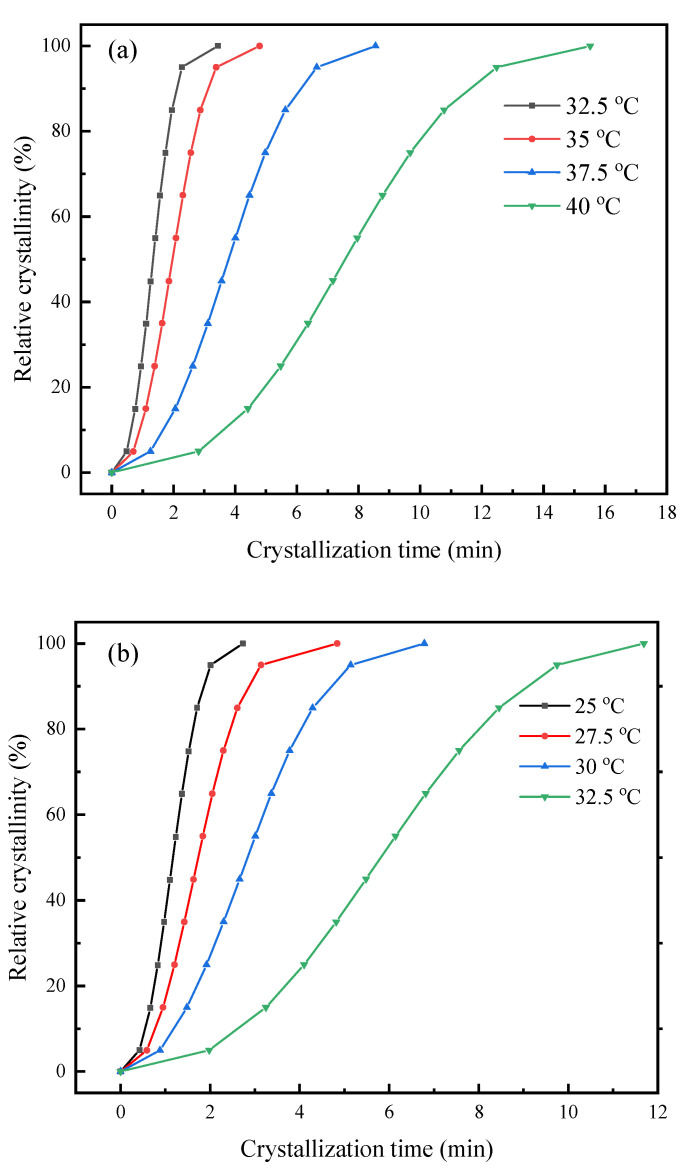
Plots of *X*_t_ versus *t* for (**a**) PHS and (**b**) PHSF5.

The isothermal melt crystallization kinetics study of the PHS and PHSF copolyesters was further investigated with DSC. Figure 4 shows the variation in relative crystallinity (*X*_t_) with crystallization time (*t*) for PHS and PHSF5 at different *T*_c_ values. As illustrated in Figure 4, for the same sample, the crystallization time prolonged with increasing *T*_c_, indicating a slow crystallization rate. At the same *T*_c_ of 32.5 °C, the crystallization time of PHSF5 was remarkably longer than that of PHS, indicating that the copolyester crystallized more slowly than PHS. The above results implied that both the small degree of supercooling and the slightly increased HF unit were the two main reasons for the longer crystallization time. PHSF10 showed a similar variation trend, as illustrated in Figure S1 in the Supporting Information.

**Figure 5:**
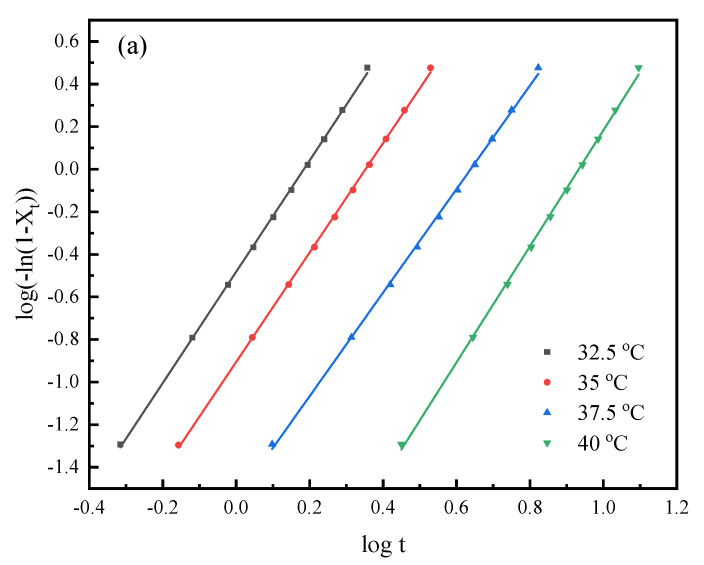

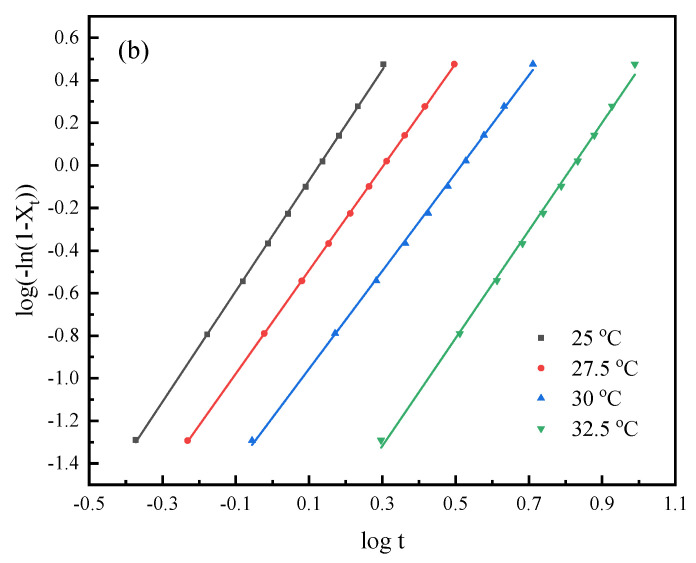
The related Avrami plots for (**a**) PHS and (**b**) PHSF5.

The well-known Avrami equation was utilized to analyze the overall isothermal melt crystallization kinetics of the PHS and PHSF copolyesters. The relationship between *X*_t_ and *t* may be described by the Avrami equation as follows:

(2) $$1\;-{\;X}_{t}{=e}^{{-kt}^{n}}$$

where *k* is the crystallization rate constant related to both nucleation and growth-rate parameters, and *n* is the Avrami exponent depending on the nature of nucleation and the growth geometry of the crystals [30,31]. Figure 5 displays the related Avrami plots for PHS and PHSF5, while the Avrami plots of PHSF10 are illustrated in Figure S2 for brevity. The Avrami parameters *n* and *k* were calculated from the slopes and intercepts of the corresponding plots and are summarized in Table 3. The *n* values slightly varied from 2.3 to 2.7 for both PHS and PHSF copolyesters, indicating that the crystallization mechanism remained unchanged for all samples. The crystallization mechanism of the PHS and PHSF copolyesters may correspond to an athermal nucleation and three-dimensional spherulite growth [32], which needs further investigation on the basis of optical microscopy observation. On the one hand, the *k* values gradually decreased with an increase in *T*_c_ for both PHS and PHSF copolyesters, suggesting again that the decreased degree of supercooling reduced the crystallization rate. On the other hand, the *k* values became smaller in the copolyesters with an increase in the HF unit content at the same *T*_c_, confirming again that the higher the HF unit content, the slower the crystallization rate. In addition, crystallization half-time (*t*_0.5_), the time to achieve 50% of the final crystallinity, was calculated by the following equation:

(3) $$t_{0.5}=(\frac{\ln2}{k}{)}^{1/n}$$

The influence of *T*_c_ and the HF unit content on the crystallization rate may further be concluded from the *t*_0.5_ data listed in Table 2, similar to those from the *k* values.

### 3.5. Melting Behavior and Equilibrium Melting-Point Study

**Figure 6:**
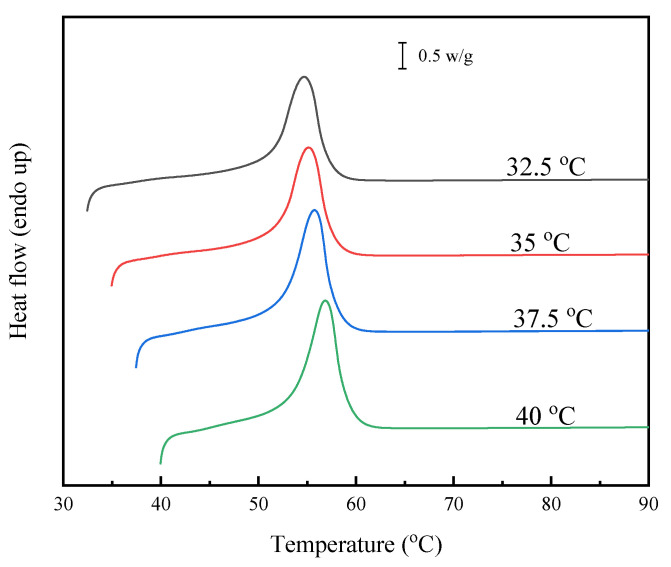
Melting behavior of PHS after crystallizing at the indicated temperatures.

The subsequent melting behavior was further studied with DSC for PHS and its copolyesters after crystallizing at indicated temperatures. As shown in Figure 6 and Figure S3 in the Supporting Information, both PHS and its copolyesters displayed similar melting behavior. Unlike the melting behavior after the nonisothermal melt crystallization (Figure 3b), only one endothermic peak was observed after the isothermal melt crystallization at the indicated temperatures. Furthermore, the *T*_m_ gradually increased with the increasing of the *T*_c_. During the heating process after the isothermal melt crystallization, the melting, recrystallization, and remelting phenomenon did not occur, which should be attributed to the more perfect crystals formed through this crystallization process.

**Figure 7:**
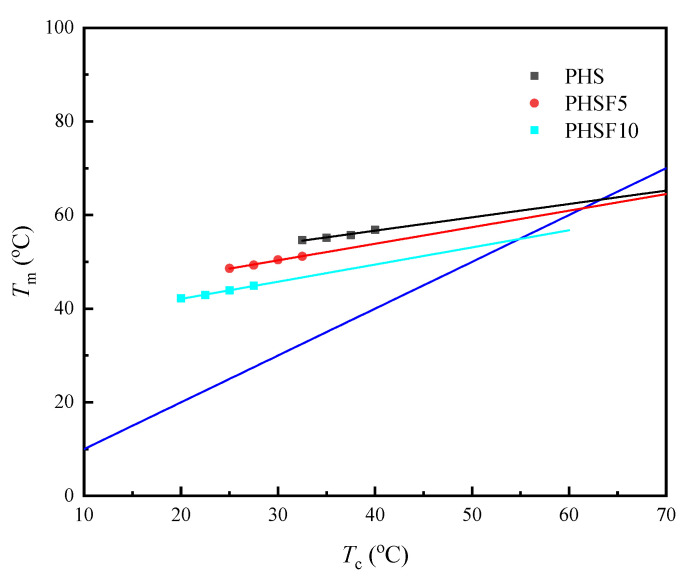
Hoffman−Weeks plots of PHS, PHSF5, and PHSF10.

The classical Hoffman–Weeks equation was used to determine the equilibrium melting-point (*T*_m_^o^) values of PHS and PHSF copolyesters as follows:

(4) $$T_{m}{=\eta T}_{c}+(1\;-{\;\eta )T}_{m}{}^{o}$$

where *η* is related to the stability of the crystals undergoing the melting process [33]. Figure 7 depicts the Hoffman–Weeks plots, from which the *T*_m_^o^ values for PHS, PHSF5, and PHSF10 were derived to be 63.3 °C, 61.4 °C, and 54.9 °C, respectively.

### 3.6. Thermal Stability Study

**Figure 8:**
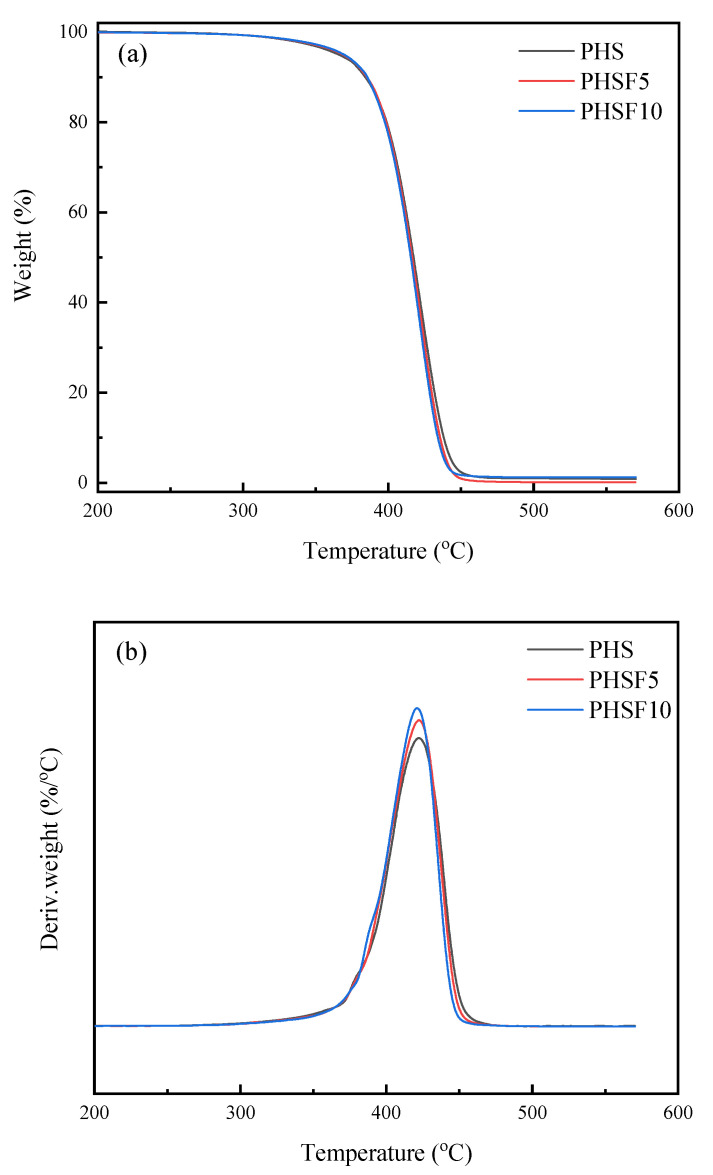
(**a**) TGA curves and (**b**) DTG curves for PHS, PHSF5, and PHSF10.

The thermal stability of the PHS and PHSF copolyesters was studied with thermogravimetric analysis (TGA). As illustrated in Figure 8, both PHS and PHSF copolyesters displayed a one-step thermal decomposition process. From parts a and b of Figure 8, the temperature at 5% weight loss (*T*_d_) and the maximum weight-loss rate (*T*_max_) were read, respectively, and are listed in Table 2. All samples showed rather high *T*_d_ and *T*_max_ values, which is indicative of the high thermal stability. For instance, the *T*_d_ slightly increased from 365.7 °C for PHS to 368.5 °C for PHSF5 and 369.2 °C for PHSF10, respectively, confirming that the presence of the HF unit retarded the thermal degradation and increased the thermal stability of PHS to some extent. However, it should be noted that the *T*_max_ values varied slightly between 421.1 and 422.4 °C for all samples, indicating that the presence of the HF unit did not show an obvious influence on *T*_max_.

### 3.7. Mechanical Property Study

**Figure 9:**
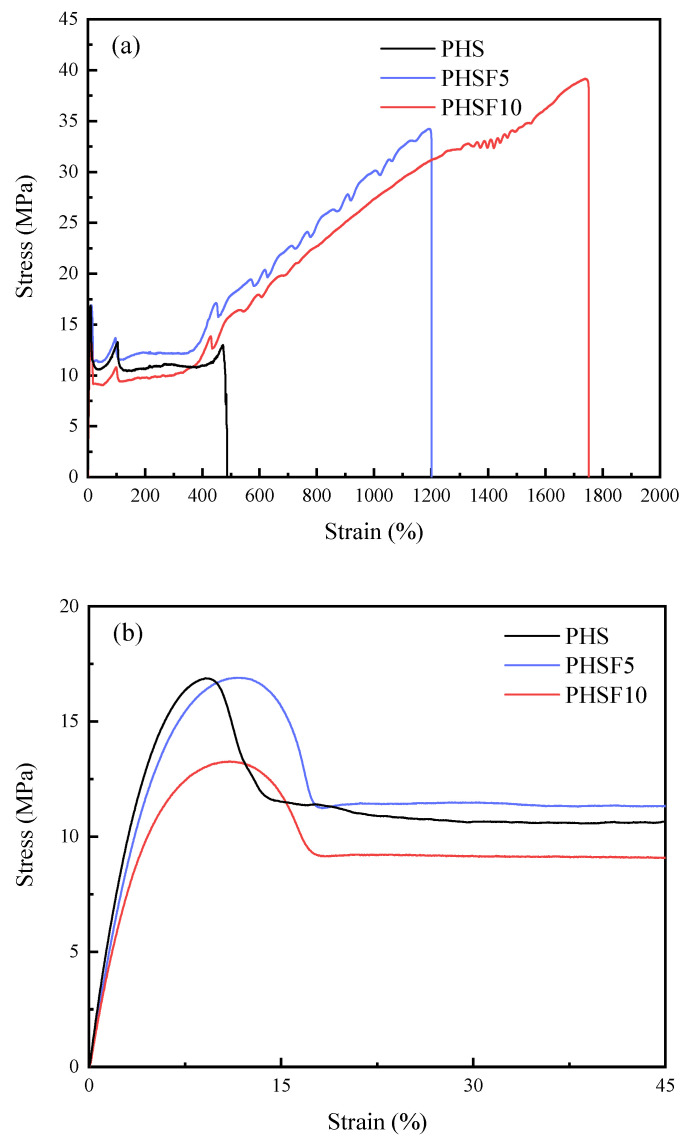
Stress–strain curves of PHS, PHSF5, and PHSF10: (**a**) whole range and (**b**) enlarged part at small strain.

The tensile mechanical property study should be of great importance from a practical application viewpoint. Figure 9 presents the stress–strain curves of PHS, PHSF5, and PHSF10. All samples showed an obvious yield behavior at the small strain region. From Figure 9, the relevant tensile mechanical property data, such as the Young’s modulus (*E*_t_), yield strength (*σ*_y_), tensile strength (*σ*_b_), and elongation at break (*ε*_b_), were determined and are summarized in Table 4. The *E*_t_ values decreased slightly from 394.7 ± 7.6 MPa for PHS to 378.2 ± 24.2 MPa for PHSF5 and 305.0 ± 6.5 MPa for PHSF10. The *σ*_y_ of PHS was 16.9 ± 1.1 MPa. The *σ*_y_ of PHSF5 was 16.9 ± 1.5 MPa, while the *σ*_y_ of PHSF5 was only 11.0 ± 0.1 MPa. Compared with those of PHS, the *σ*_b_ and *ε*_b_ values of the PHSF copolyesters significantly increased. The *σ*_b_ and *ε*_b_ values of PHS were only 12.9 ± 0.9 MPa and 498.5 ± 4.78%, respectively. In the presence of about 5 mol% of HF unit, PHSF5 displayed a *σ*_b_ of 33.4 ± 1.4 MPa and an *ε*_b_ of 1228.4 ± 94.3%, respectively, while with further increasing of the HF unit to about 10 mol%, PHSF10 displayed a *σ*_b_ of 39.2 ± 0.8 MPa and an *ε*_b_ of 1757.6 ± 6.1%, respectively. Such an improvement in the mechanical property should be related to the presence of the rigid HF unit and the decrease in crystallinity of the copolymers. As a result, the tensile mechanical property of PHS may be remarkably improved through the copolymerization method in this research, which should be of great help and importance for its further practical application as a packaging material.

## 4. Conclusions

In this research, two fully biobased PHSF copolyesters with about 5 and 10 mol% of HF unit were successfully synthesized through a two-step melt polycondensation process. Both the chemical structure and the HF composition were analyzed and determined by ^1^H NMR. PHS and PHSF copolyesters had rather high *M*_w_ values of 6.75 × 10^4^ to 9.30 × 10^4^ g/mol. The basic thermal behavior, crystal structure, isothermal crystallization kinetics, melting behavior, thermal stability, and tensile mechanical property of the PHSF copolyesters were studied in detail and compared with those of PHS. Due to the presence of a small amount of HF unit, PHSF showed a decrease in the melt crystallization temperature, melting temperature, and equilibrium melting temperature, while showing a slight increase in the glass transition temperature and thermal decomposition temperature. PHSF copolyesters displayed the same crystal structure as PHS, indicating that the HF unit should be expelled into the amorphous region of the PHS crystals. The crystallization rate became slower with an increasing crystallization temperature for both PHS and PHSF copolyesters; moreover, PHSF copolyesters crystallized more slowly than PHS. Within the investigated temperature range, the crystallization mechanism remained unchanged. In addition, PHSF copolyesters showed an improved mechanical property compared with that of PHS. For example, PHSF10 displayed a *σ*_b_ of 39.2 ± 0.8 MPa and an *ε*_b_ of 1757.6 ± 6.1%, while those of PHS were only 12.9 ± 0.9 MPa and 498.5 ± 4.78%, respectively. In brief, through an easy copolymerization method, the thermal and mechanical properties of PHS may be tuned to meet practical application requirements from a sustainable viewpoint.

## Figures and Tables

**Table 1:** Compositions and molecular weights of PHS and PHSF copolyesters.

Samples	SA/DMFD (mol%)	HS/HF (mol%)	*M*_n_ (g/mol)	*M*_w_ (g/mol)	PDI
PHS	100/0	100/0	4.06 × 10^4^	6.75 × 10^4^	1.66
PHSF5	95/5	94.9/5.1	4.88 × 10^4^	9.30 × 10^4^	1.91
PHSF10	90/10	89.9/10.1	5.16 × 10^4^	9.30 × 10^4^	1.80

**Table 2:** Basic thermal behavior data of PHS and PHSF copolyesters.

Samples	*T*_d_	*T*_max_	*T*_g_	*T*_m_	Δ*H*_m_	*T*_cc_	Δ*H*_cc_	*T*_m_^o^
	(°C)	(°C)	(°C)	(°C)	(J/g)	(°C)	(J/g)	(°C)
PHS	365.7	422.2	−48.8	53.4	68.3	26.1	66.2	63.3
PHSF5	368.5	422.4	−43.9	47.1/50.1	50.9	15.9	49.4	61.4
PHSF10	369.2	421.1	−43.1	38.4/43.4	43.1	2.4	37.6	54.9

**Table 3:** Summary of related Avrami parameters for PHS and PHSF copolyesters.

Samples	*T*_c_ (°C)	*n*	*k* (min^−*n*^)	*t*_0.5_ (min)
PHS	32.5	2.6	3.29 × 10^−1^	1.32
	35	2.6	1.24 × 10^−1^	1.95
	37.5	2.4	2.81 × 10^−2^	3.74
	40	2.7	2.87 × 10^−3^	7.49
PHSF5	25	2.6	4.67 × 10^−1^	1.16
	27.5	2.4	1.84 × 10^−1^	1.73
	30	2.3	6.53 × 10^−2^	2.79
	32.5	2.5	8.38 × 10^−3^	5.72
PHSF10	20	2.3	1.11 × 10^−1^	2.20
	22.5	2.5	2.97 × 10^−2^	3.56
	25	2.5	1.12 × 10^−2^	5.06
	27.5	2.5	2.69 × 10^−3^	8.83

**Table 4:** Summary of mechanical property data of PHS and PHSF copolyesters.

Samples	*E*_t_	*σ*_y_	*σ*_b_	*ε*_b_
	(MPa)	(MPa)	(MPa)	(%)
PHS	394.7 ± 7.6	16.9 ± 1.1	12.9 ± 0.9	498.5 ± 4.78
PHSF5	378.2 ± 24.2	16.9 ± 1.5	33.4 ± 1.4	1228.4 ± 94.3
PHSF10	305.0 ± 6.5	11.0 ± 0.1	39.2 ± 0.8	1757.6 ± 6.1

## Data Availability

The data presented in this study are available on request from the corresponding author.

## Supplementary Materials

The following supporting information can be downloaded at: https://www.mdpi.com/article/10.3390/polym15020427/s1, Figure S1: Plots of relative crystallinity versus crystallization time for PHSF10; Figure S2: The related Avrami plots for PHSF10; Figure S3: DSC heating curves after isothermal crystallization at the indicated temperatures for (a) PHSF5 and (b) PHSF10.
