# Supplementary material for: Synthesis, Thermal and Mechanical Properties of Fully Biobased Poly (hexamethylene succinate-co-2,5-furandicarboxylate) Copolyesters
Source: Polymers (Basel). 2023 Jan 13;15(2):427. doi: 10.3390/polym15020427 (PMC9866186; doi:10.3390/polym15020427)
Supplement: Supplementary file 1 [file polymers-15-00427-s001.zip › polymers-2128557-supplementary.pdf]

polymers

Supplementary Materials

Synthesis, Thermal and Mechanical Properties of Fully Biobased Poly (hexamethylene succinate-*co*-2,5-furandicarboxylate) Copolyesters

Chengqian Wang, Mingkun Chen, Zhiguo Jiang * and Zhaobin Qiu *.

State Key Laboratory of Chemical Resource Engineering, Beijing University of Chemical Technology, Beijing 100029, China
* Correspondence: jiangzg@mail.buct.edu.cn (Z.J.); qiuzb@mail.buct.edu.cn (Z. Q.)

Materials

The monomers SA, HDO, and DMFD were purchased from Tianjing Fuchen Chemical Solvent Factory, Shanghai Macklin Biochemical Co. Ltd., Ningbo Institute of Materials Technology and Engineering, Chinese Academy of Sciences, respectively. The catalyst TBT was obtained from Beijing Changping Jingxiang Chemical Factory. All chemicals were used as received without any purification.

Synthesis of PHSF copolyesters

PHS and PHSF copolyesters were synthesized through a two-step transesterification/esterification and polycondensation method, as illustrated in Scheme S1. First, an appropriate amount of monomers at a diol (HDO)/diacid or diestser (SA+DMFD) ratio of 1.2 and 1 mL (1 wt%) of TBT solution were charged into a three-necked round-bottom flask equipped with a mechanical stirrer, a condensing device, and a nitrogen inlet. In order to remove O_2 from the device, the flask was purged with nitrogen three times. During the next polycondensation process, a high-vacuum of 50-100 Pa was applied slowly over a time of 15-20 min to prevent the sublimation of oligomer molecules. Then, the polycondensation reaction was conducted at 235 °C for about 3-5 h.

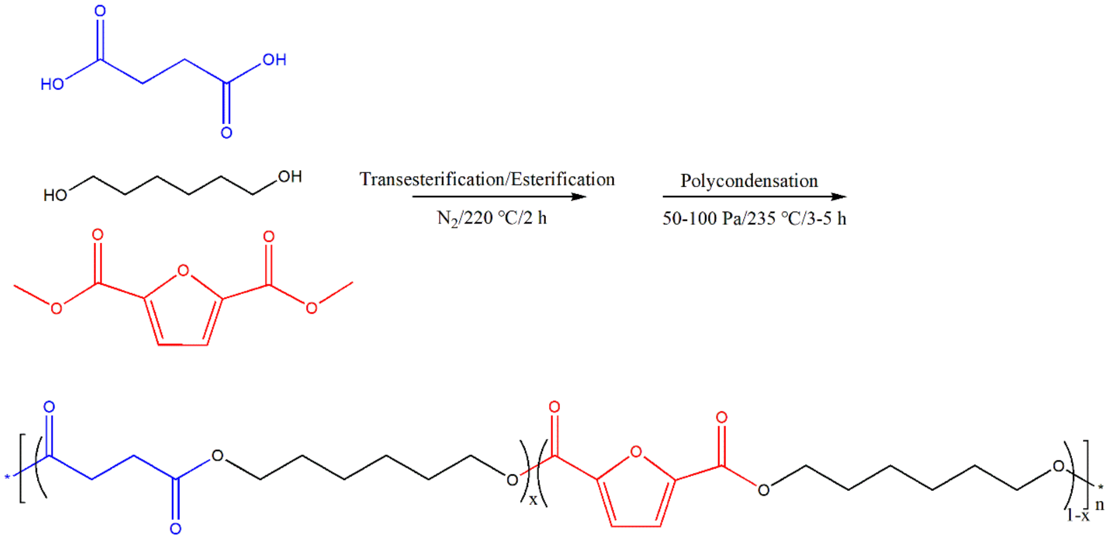

Scheme S1. Synthesis route of PHSF copolyesters.

Characterization

The number-average molecular weight (M_n), weight-average molecular weight (M_w), and polymer dispersity index (PDI) were measured at 25 °C by a Gel Permeation Chromatography (GPC, GPC Waters 1515), using THF as the mobile phase at a flow rate of 1 mL/min. Polystyrene standards with narrow polydispersity were used for calibration.

The Hydrogen Nuclear Magnetic Resonance (^{1}H NMR) spectra of the PHSF copolyesters were recorded on a Bruker AV-600 (600 MHz) with $CDCl_3$ used as the solvent.

The thermal behavior of PHSF copolyesters was recorded by using Differential scanning calorimetry (DSC) (TA Instruments Q100). First, the samples were heated to 100 °C and kept for 3 min to erase thermal history, then quenched to −70 °C at a rate of 60 °C/min, and afterwards they were reheated to 100 °C at 20 °C/min to record the T_g values. For the nonisothermal melt crystallization behavior, the samples were cooled to −70 °C at 10 °C/min after erasing thermal history, then reheated to 100 °C to obtain the T_{cc} and T_m values.

The isothermal melt crystallization study was also investigated. The samples were quenched to the indicated temperature (T_c) at 60 °C/min and then reheated to 100 °C at 10 °C/min after finishing the isothermal crystallization.

The thermal stability was measured by Thermogravimetric analysis (TGA) (TA Instruments Q50) from room temperature to 500 °C at a heating rate of 20 °C/min under nitrogen atmosphere.

Wide-angle X-ray diffraction (WAXD) (Rigaku Model Max2500 X-ray diffractometer) was used to investigate the crystal structure from 5 ° to 50 ° at 5 °/min. The samples were crystallized at 32 °C for 10 h.

Tensile tests were performed by a universal tensile testing machine (UTM5205XHD) at 20 mm/min at room temperature. The specimens were prepared by hot-press molding at 70 °C. At least, three values were collected to obtain the average data.

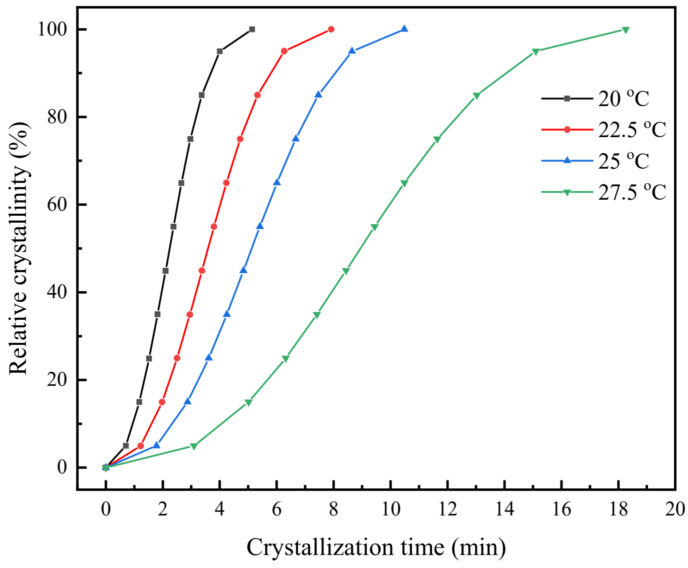

Figure S1. Plots of relative crystallinity versus crystallization time for PHSF10.

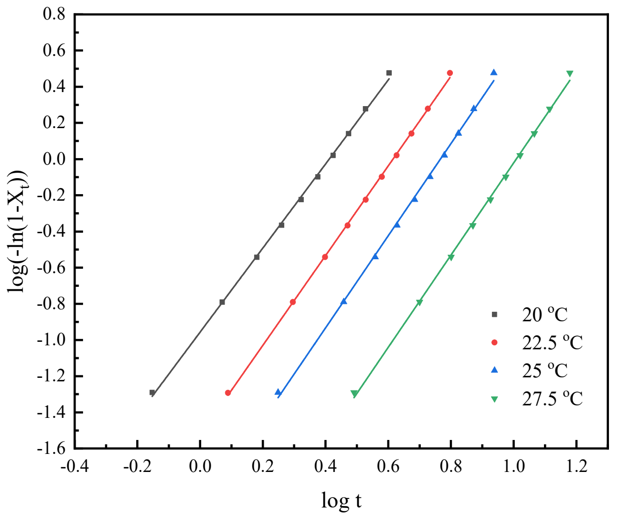

Figure S2. The related Avrami plots for PHSF10.

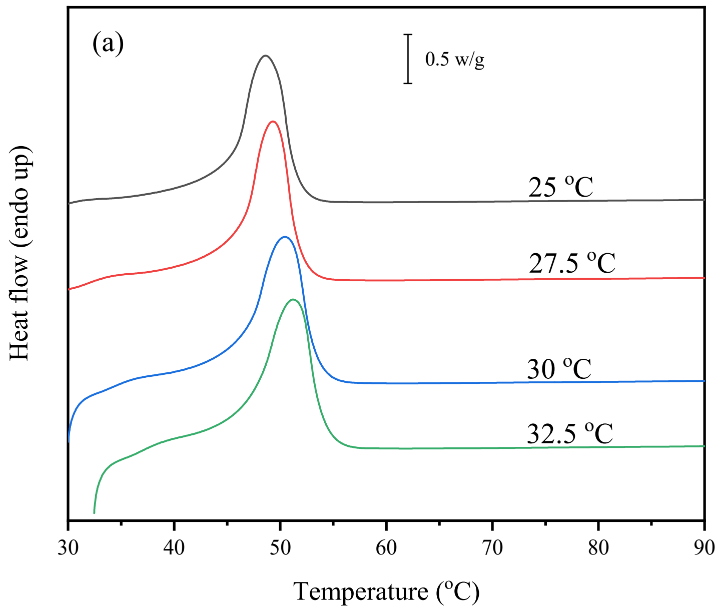

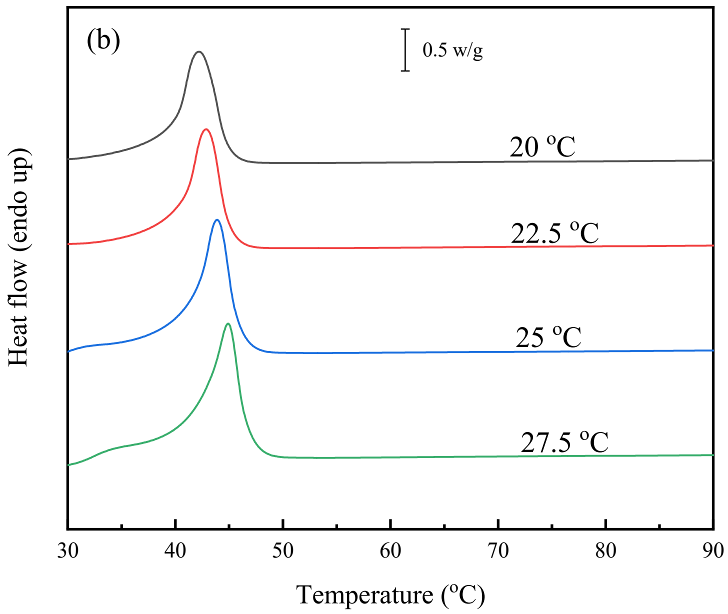

Figure S3. DSC heating curves after isothermal crystallization at the indicated temperatures for (a) PHSF5 and (b) PHSF10.
